# Validity and Reliability of the Dental Neglect Scale in German

**DOI:** 10.3390/dj13050225

**Published:** 2025-05-21

**Authors:** Katharina Marilena Weil, Theresa Marie Weßlau, Laura Agnes Ingrid Magerfleisch, Hannah Tröger, Lisa Irmscher, David Bantel, Clara Theres Meyer-Probst, Katja Petrowski, Hendrik Berth

**Affiliations:** 1 Research Group Medical Psychology and Medical Sociology, Division of Psychological and Social Medicine and Developmental Neurosciences, Medical Faculty Carl Gustav Carus, Technische Universität Dresden, Fetscherstr. 74, 01307 Dresden, Germany; katharina_marilena.weil@mailbox.tu-dresden.de (K.M.W.); theresa_marie.wesslau@mailbox.tu-dresden.de (T.M.W.); laura_agnes_ingrid.magerfleisch@mailbox.tu-dresden.de (L.A.I.M.); hannah.troeger@mailbox.tu-dresden.de (H.T.); lisa.irmscher@ukdd.de (L.I.); david.bantel@hin.ch (D.B.); 2 Clinic of Operative Dentistry, Medical Faculty Carl Gustav Carus, Technische Universität Dresden, Fetscherstraße 74, 01307 Dresden, Germany; claratheres.meyer-probst@ukdd.de; 3 Department of Medical Psychology and Medical Sociology, University Medical Center of the Johannes Gutenberg-University Mainz, Duesbergweg 6, 55131 Mainz, Germany; kpetrows@uni-mainz.de

**Keywords:** dental neglect scale, reliability, validity, oral self-care, oral health

## Abstract

**Background/Objectives:** The Dental Neglect Scale (DNS) is known and used to evaluate and assess adult behaviors and attitudes related to oral self-care and oral-health. In English-speaking countries, the DNS has been used in many studies. At the moment, there is no validated version of the DNS that is available in the German language. The aim of this study is to validate a German version of the DNS to evaluate and assess the oral health and behavior among the German population. **Methods:** The study population consisted of N = 311 German adults (180 female, 130 male, 1 diverse) aged from 18 to 90 years from a dental clinic in Germany. The participants answered a questionnaire. In addition to questions on their socio-demographic background and the DNS, the survey included more standardized instruments relating to preventive behavior, dental anxiety, and oral health. **Results:** The DNS was translated into German. An explanatory factor analysis was conducted, which supported the unifactorial structure of the scale. The following results were obtained for the reliability of the resulting DNS: Cronbach’s Alpha = 0.710, McDonald’s Omega = 0.711, and Mean = 25.63 (SD = 4.02, Range 12–30). Convergent and divergent validity were demonstrated through associations between the DNS and various oral health scales, and by differences between genders and age groups. **Conclusions:** This study confirms the quality of the criteria of the DNS for German adults. The DNS is a convincing instrument that is used for epidemiological studies in the field of dentistry and psychology. Further validation with other samples should be conducted.

## 1. Introduction

The Dental Neglect Scale (DNS) creates a reliable connection between the neglect of dental health and care and other factors that relate to the respondents’ cultural, social, and attitudinal backgrounds [1]. The characteristics of dental neglect are reflected in a lack of oral hygiene, which is defined as the failure to brush one’s teeth, maintain interdental space care, use mouthwash, maintain a healthy diet, and attend regular professional teeth cleaning and control appointments at a dental clinic. In addition, individuals who exhibit dental neglect are unable to pursue dental treatments [2] and lack awareness of their oral health [3]. Dental neglect occurs when necessary dental treatments are neglected, leaving conditions such as tooth decay or periodontitis untreated. This can lead to pain, infection, and dysfunction, which in turn impair important aspects of daily life such as speaking, eating, learning, and age-appropriate development (especially for children). The Dental Neglect Scale addresses all of these aspects. As a result, it is an important tool for examining dental care and neglect of dental health. The scale examines individuals’ sense of responsibility for their dental care, dental visits, and appreciation of dental health [4,5].

### Dental Neglect Scale

The DNS has been translated and used in various contexts in recent years. The scale has been used particularly frequently in English-speaking countries. The psychometric properties of the scale have been examined in adults [6,7], adolescents [8,9], children [10,11,12], and students from diverse academic disciplines [13,14]. The scale was originally developed and validated for Australian children to provide an assessment of dental neglect based on their parents’ responses [1]. The version for children contains seven items, while the version for adults contains six items [4,5]. Two factors were identified in studies of young adults [4] and adolescents [8]. In the Norwegian [7] and Romanian [6] populations, studies identified a single factor. These investigations thus indicate the need for further psychometric evaluation. Another possible limitation of studies on the DNS is that much less research has been conducted on the psychometric properties of this instrument when used for adults than when it is used for children [10,12]. Further studies demonstrate correlations between high DNS values and the severity of caries, missing teeth [15], few visits to the dentist [8,13,16], and unsatisfactory assessments of one’s own dental health [6]. The Romanian variant [6] refers to sample comparisons of American adolescents [8], Indian adolescents [9], young Nepalese population groups [17], and young educated Indians [14]. The average values for American adolescents are 13.20 ± 3.80 [8], for Indian adolescents they are 10.18 [9], for young Nepalese adults they are 15.61 ± 2.40 [17], and for young educated Indians they are 19.71 ± 3.94 [14]. Cronbach’s Alpha coefficients such as 0.71 [5], 0.60 [8], 0.65, and 0.57 [7] were determined in various samples. These indicate an acceptable consistency. The DNS has been shown to be a good predictor of dental visit patterns [8] and provides an estimate for the clinical assessment of the severity of caries lesions [13]. Various studies have shown that there is a connection between dental neglect and the impact of dental problems on indivudals’ quality of life [4,5,17,18,19]. In terms of gender differences, there are studies that have found no differences [8,15]. Other studies indicate that males in particular show deficits in their dental health and corresponding behavioral measures [4]. This question is addressed in our research work in order to substantiate one of these two statements. Considering that DNS has already proven to be a useful tool for epidemiologic investigations in preventive dentistry in other countries [4,5,6,7], we decided to test it in German adults as well. At the present time, there is no similar instrument to the DNS that is valid for the purpose of assessing the German population.

## 2. Materials and Methods

### 2.1. Process of Translating and Adapting the Scale into Questionnaire

The procedure of translating the scale was a multi-step process that was based on the common procedures for the cross-cultural adaption and validation of questionnaires [20]. Initially, the English version was translated into German by bilingual German dentists. Subsequent to this, the translated German version was translated back into English by two additional dentists. Correct and contextual translation is of importance, as the interviewed persons should be able to easily understand and answer the questionnaire. The final German version of the DNS was pre-tested on a sample of N = 20 medical students at the Technische Universität Dresden, Germany, to ensure their comprehension of the questionnaire. As result of the pre-test, no changes in the scale were made.

### 2.2. Ethical Consideration

This study was conducted in accordance with the Declaration of Helsinki. The study protocol was approved by the Ethics Committee of the Technische Universität Dresden, Germany (SR-EK-441102022/Dresden, 8 November 2022).

### 2.3. Design and Data Collection

This study was conducted in a dental clinic in Germany during the months of September and October 2022. Participant recruitment took place at the MVZ–üBAG Dres. Weßlau & Kollegen in Bernau, Germany. Patients were approached immediately before or after their dental treatment and informed about this study. Upon providing written informed consent, they were invited to complete the questionnaire. The survey was conducted anonymously, and all data were handled with strict confidentiality. No financial compensation was provided for the participants.

### 2.4. Measures

This study was conducted using questionnaires.

**The Dental Neglect Scale (DNS)** is used to measure the behaviors and attitudes of German adults with regard to dental care, dental health, and neglect of dental health [5]. There are six items, which are rated on a scale from 1—*definitely no* to 5—*definitely yes*. One of the items is evaluated in reverse: “*I need dental care but I put it off*”. For this item, the response scale was inverted, with the coding ranging from 5—*definitely no* to 1—*definitely yes*. Examples of other items are: “*I keep up my home dental care*” and “*I brush as well as I should*”. The total score is the sum of the six items. The score rages between 6 and 30. The lower the score, the higher the dental neglect [5,8]. With regard to the reliability of this study, the Cronbach’s α was 0.710 and McDonald’s ꞷ was 0.711. Both values demonstrate acceptable internal consistency.

**The Oral Health Impact Profile (OHIP-5)** is used to assess the impact of dental problems/dental prosthesis problems on the oral health-related quality of life of adults. The original English version comprises 49 questions [21]. Since its development, further short versions have been developed. These include the first German version [22]. The OHIP-5 [23] includes five items that evaluate the respondent’s perception of well-being in their mouth area, ranging from 1—*never* to 5—*very often*. Questions are asked as to whether there are problems chewing food, whether the taste of food has changed, whether there is pain in the mouth, whether the appearance of the teeth/prosthetic teeth causes discomfort, and whether problems in the mouth restrict everyday activities. High values show a stronger influence of the respondent’s dental health on their quality of life. The reliability of this study was determined to be satisfactory, with a Cronbach’s α = 0.797 and a McDonald’s ꞷ = 0.804, indicating good reliability.

**The L-1** scale is a short scale used to measure general life satisfaction [24]. It includes the question “All in all, how satisfied are you with your life at the moment?” There are 11 possible answers ranging from *not at all satisfied* to *completely satisfied*.

**The Brief Symptom Inventory (BSI-18)** is a tool designed to assess the perceived impairments caused by various symptoms experienced over the previous seven days, providing an assessment of how the respondent feels [25]. Symptoms such as fainting or dizziness, heart and chest pain, and even anxiety are assessed. For each of the 18 items, participants can choose between five possible answers ranging from 1—*not at all* to 5—*very strongly*. A higher total score indicates greater psychological distress. BSI-18 is a very short and reliable instrument for assessing the respondent’s psychological stress [25]. In this study with a value of 0.907, Cronbach’s α indicates very good reliability. The McDonald’s ꞷ = 0.909.

**The Dental Anxiety Scale (DAS)** was first introduced by Corah in 1969 and nowadays is frequently used to assess dental anxiety in patients [26,27,28,29]. The scale comprises four questions relating to situations before and during dental treatment. The questions are to be answered on a scale from 1—*low anxiety* to 5—*high anxiety*. The score is the sum of the answers and ranges between 4 and 20. A score of 15 (cut-off value) indicates “proven dental anxiety”, scores between 13 and 15 indicate “some anxiety”, and a score below 13 indicates “little to no anxiety”. The re-test reliability of the DAS was determined to be rtt = 0.86 [26]. In this study, the Cronbach’s α value of 0.827 indicated good reliability. The McDonald’s ꞷ = 0.827, demonstrating adequate internal consistency.

**The DMF Index** has been used in dentistry for over 70 years to provide an overview of decayed, missing, and restoratively treated teeth (DMF_T) or tooth surfaces (DMF_S) in an adult dentition [30]. The letter D stands for decayed tooth, the letter M for missing tooth, and F for filled tooth. If the letters are capital letters, it indicates an adult dentition with a maximum of 28 teeth, as the wisdom teeth are not usually taken into account. In a primary dentition, the index is used with small letters—dmf_t. The DMF_S index refers to surfaces. In this index, the anterior teeth are divided into four surfaces (facial, lingual, mesial, amd distal) and the posterior teeth are divided into five surfaces (facial, lingual, mesial, distal, and occlusal). The total score ranges from 0 to 148 points if the third molars are included in the analysis. Assuming an adult dentition of 28 teeth, the score ranges from 0 to 128. Higher values indicate more damage to the teeth. The index is designed to record changes related changes to caries; however, if a tooth is lost due to trauma or periodontitis, it is not included in the index [18].

**The Periodontal Screening Index (PSI)** is measured using a WHO probe. This examination forms part of a routine dental examination. It is an assessment of periodontal health [31,32]. When measuring the PSI, the dentition is divided into sextants (third molars are not included). At the tip of the WHO probe is a small ball with a diameter of 0.5 mm, and the probe is provided with a black color scale in the range of 3.5–5.5 mm. The probe is used to probe six sites per tooth: the mesiobuccal, buccal, distobuccal, mesiooral, oral, and distooral sites. There is a code of 0–4, with 0 indicating a healthy periodontium, 1–2 indicating gingivitis, and 3–4 indicating periodontitis [33].

**Preventive behavior items** were developed specifically for this study. The items assess preventive behavior and form a self-constructed questionnaire. The first item addresses the responent’s daily tooth brushing frequency, with response options ranging from *once* to *four times per day*. The second item assesses the frequency of dental visits, with options ranging from *never* to *more than four times per year*. The third item concerns the annual frequency of tartar removal, and the fourth item assesses the frequency of professional tooth cleaning per year. The response options for both range from *never* to *four times per year*. The internal consistency of this metric was not calculated, as the items were intended to capture distinct dimensions of preventive behavior rather than a unidimensional construct.

### 2.5. Sociodemographic Variables

The sociodemographic variables employed in this study pertain to the following domains: the variables encompassed by this study included gender (female, male, and diverse), age, graduation, employment, children, and family status or partnership. The participants’ preventive behavior was measured on the basis of four questions regarding the frequency of their tooth brushing, dentist visits, tartar removal, and professional tooth cleaning.

### 2.6. Data Analysis

Statistical analysis were conducted using IBM SPSS 30. Standard deviations and skewness and kurtosis indicators are shown. The Cronbach’s α is also shown, and was obtained by deleting one item. Furthermore, the reliabilities (Cronbach’s α, McDonald’s ꞷ) were calculated. Correlations between the various measures were also examined. The chi-square test and the test statistic were used to calculate group differences. The construct validity of the scale was tested by performing an exploratory factor analysis (EFA) and a confirmatory factor analysis (CFA) using RStudio version 2024.12 [34] and the ‘Lavaan’ package version 0.6-19 [35]. For these analyses, the sample was randomly split into two subsamples while controlling for a similar distribution of age and gender. The first subsample (n = 160; M_age_ = 51.0; n_male_ = 94) was used for the EFA, and the second subsample (N = 151; M_age_ = 51.7; n_male_ = 86) was used for the CFA.

To interpret the results of the CFA, the model’s fit was tested using the following coefficients: the χ^2^ (Chi-squared), the comparative fit index (CFI), the root mean square error of approximation (RMSEA), the standardized root mean square residual (SRMR), and the Tucker–Lewis Index (TLI). The indices for the model fit were considered good or acceptable, following common recommendations. For the model to be considered a good (or acceptable) fit, the CFI and TLI should be 0.95 (0.90; Hu & Bentler, 1999 [36]), the ratio of χ^2^/df should be smaller than 2 (3), the RMSEA should be <0.05 (<0.08), and the SRMR should be <0.05 (<0.10) [37].

The required sample size was determined using G*Power 3.1.3 [38]. For example, for the calculation of Pearson correlations (two-tailed) with a significance level of α = 0.05 (effect size q = 0.5; power (1−β) = 0.95), a sample size of at least N = 214 persons is necessary. The sample size in this study (N = 311) was sufficient for all statistical methods that were used.

### 2.7. Hypotheses

**Hypothesis** **1:** *We assume that DNS has a unifactorial structure based on the abovementioned literature [6,7].*

**Hypothesis** **2:** *We suspect a connection between DNS and mental health as well as dental health [5,15]. We hypothesize that individuals who score lower on the DNS (thus who have higher dental neglect) have higher scores on the BSI-18 (Brief Symptom Inventory) and DAS (Dental Anxiety Scale).*

**Hypothesis** **3:** *We assume that there are differences with regards to gender, education, and age groups. Gender-specific differences have been found in previous studies that used the DNS [4,15]. We suspect that the same pattern can be found in our study. Does school graduation have an impact on dental neglect? Do older adults show lower scores on the Dental Neglect Scale compared to younger people?*

## 3. Results

### 3.1. Sociodemographic Characteristics

The sample that was analyzed consisted of 311 participants (mean age = 51.34; SD = 18.82; range: 18–90 years. There were 180 female participants (57.9%), 130 male participants (41.8%), and one diverse participant (0.3%). A total of 233 participants (71.7%) stated that they had a child or children (median = 1.0; S.A. = 0.46) and 84 participants (27.0%) were childless. N = 226 participants (72.7%) lived in a partnership, while 76 participants (24.4%) lived alone (median = 1.0; SD = 0.46). A total of 52 participants (16.7%) had an elementary school certificate, 135 participants (43.4%) had a secondary school leaving diploma/certificate, 106 participants (34.1%) had A-Levels/a high school diploma, 4 participants (1.3%) had a certificate from a special school, 8 participants (2.6%) had no certificate, and a further 6 (1.9%) had another school certificate (median = 2.0; SD = 0.99). A total of 180 of the respondents (57.9%) were employed, 96 (30.9%) were retired, 6 (1.9%) were housewives/husbands, 6 (1.6%) were unemployed, 18 (5.8%) were otherwise employed, and 5 (1.4%) participants did not respond to the survey.

### 3.2. Descriptive Analysis

The means, standard deviations, skewness, and kurtosis indicators are represented in Table 1. The Cronbach’s α is also shown, and was obtained by deleting one item. For items two and six, the kurtosis values are above the value of 3 [39], which indicates that not all items have a normal distribution. The Cronbach’s α = 0.710 and McDonald’s ꞷ = 0.711 indicate good internal consistency. The average total score of the DNS-GER was M = 25.63 (SD = 4.02). A comparison to other studies shows that German participants indicate a high level of dental care. The values vary between 10.18 and 19.71 [18,23,25]. The Romanian variant displays a comparable level, exhibiting an average value of 25.29 [6]. In the present study, the items “I consider my dental health to be important” (M = 4.71; SD = 0.68) and “I receive the dental care I should” (M = 4.62; SD = 0.87) achieved the highest values.

### 3.3. Exploratory Factor Analysis

The results of the exploratory factor analysis, using the principal component analysis with varimax rotation, suggested that one factor accounted for 46.4% of the explained variance. Table 2 shows the standardized loadings based upon a correlation matrix.

### 3.4. Confirmatory Factor Analysis

Due to the non-normal distribution of the data, the confirmatory factor analysis was conducted using the robust Maximum Likelihood Robust (MLR) estimator. Additionally, bootstrapping with 2000 resamplings (95% confidence intervals) was applied to account for non-normality and ensure the robustness of the results. The obtained model-fit indices, as shown in Table 3, are all considered good or at least acceptable; therefore, the model can be considered as valid.

### 3.5. Convergent Validity

**Figure 1:**
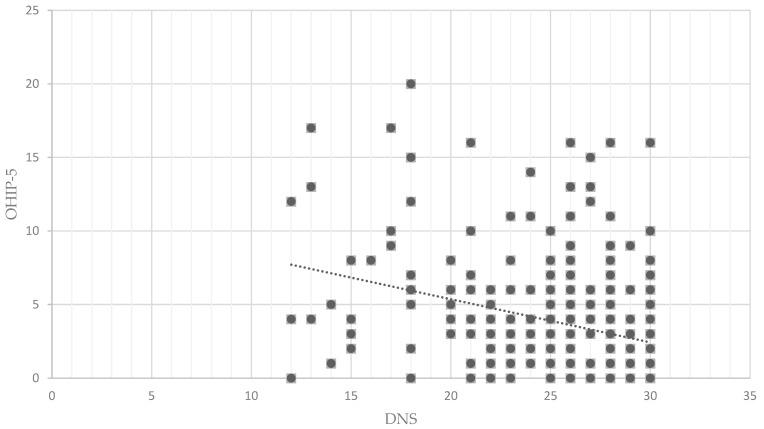
Scatterplot of the Dental Neglect Scale (DNS) and the Oral Health Impact Profile (OHIP-5).

The convergent validity describes how well the DNS agrees with other instruments, such as the BSI-18, OHIP-5, DMF_T, DMF_S, PSI, and DAS. The DNS achieved values with weak to moderate negative correlation with the BSI-18 (r = −0.283), OHIP-5 (r = −0.299), and DAS (r = −0.278, all *p* < 0.001) (Table 4). The higher the psychological stress, the less attention is paid to dental health (low DNS values). The more a person neglects their dental health (low DNS value), the worse the person assesses their oral quality of life to be (high OHIP-5 value, see Figure 1). With regard to the DAS, the negative correlation reflects that the more fear a person has of the dentist (high DAS value), the less their dental health is neglected (high DNS value). The DMF_T and DMF_S achieved very weak positive correlations (r ≈ 0.13) and statistically significant correlations with *p* < 0.05 (*p* = 0.024 and *p* = 0.020). The more a person neglects their dental care (low DNS value), the higher their DMF values. The DNS and PSI showed no significant correlation (r = 0.066, *p* = 0.290).

### 3.6. Differences Between Gender, Education and Age Groups

On the basis of the DNS values, the sample was divided into two groups of approximately equal size with a DNS value ≤ 27 (N = 158, 52.8%) and a DNS value greater than 27 (N = 147, 48.2%), respectively (Table 5). This study revealed no statistically significant differences between the male and female participants with regard to high or low DNS values (*p* = 0.287). The tests on age resulted in a chi-square = 38.77, *p* < 0.001 ** and a Phi = 0.357. These values indicate a highly significant correlation between age and group membership. Older people (50+) have a higher probability of 64.3% of belonging to the high group >27 (low dental neglect). Younger people (up to 49) were more likely to belong to the lower group ≤27 (high dental neglect, 71.5%). The present study found no statistically significant correlations between the level of education and dental negligence (chi-square = 0.552, *p* = 0.457). With regard to the oral health-related variables (PSI, DMF_T, DMF_S, and DAS), significant differences were identified between individuals with high and low levels of dental neglect. The individuals with low scores (high dental neglect) had slightly lower PSI scores than those with high scores (lower dental neglect). The observed difference was deemed to be of significance, with a *p* = 0.021; however the effect size was considered to be negligible (d = −0.290). The participants with high values had significantly higher values for DMF_T (*p* = 0.006) and DMF_S (*p* = 0.005). The effect size was determined to be small to medium (d = −0.332). The individuals with low values exhibited significantly higher levels of dental anxiety (*p* < 0.001 **, d = 0.548).

### 3.7. DNS and Preventive Behavior

Our survey revealed that participants with a low DNS score were more likely to brush their teeth only once a day (11.9%) than those with a high DNS score (4.0%) (Table 6). The individuals in the high DNS group most frequently brushed twice a day (39.3% vs. 35.0% in the lower group). Brushing three times a day was also slightly more common in the group with a high DNS score (5.0% vs. 3.6%). A significant difference between the low vs. high DNS score groups was shown regarding tooth brushing frequency (*p* = 0.003). The effect size was moderately strong (phi-coefficient = 0.216). A significant relationship was identified between the respondents’ group (low vs. high DNS score) and their frequency of visits to the dentist (*p* = 0.010, phi-coefficient = 0.224). Individuals with a low DNS score were more likely to never go to the dentist (4.0%) than people with a high DNS score (0.3%). The participants with a high DNS score were shown to be more likely to visit the dentist twice (20.7% vs. 15.7%) or three times a year (3.3% vs. 2.0%) compared to the participants with a low DNS score. The participants with a low DNS score stated that they visit the dentist more than four times a year (4.7% vs. 3.0%). A significant difference was found between participants with a low vs. high DNS score regarding their frequency of scaling (*p* = 0.006): 12–2% of the participants with a lower DNS score stated that they had never had tartar removed. The participants with a higher DNS score were shown to be more likely to have tartar removed twice a year (16.9% vs. 12.9%). Generally, tartar removal three times a year or more was rare, but occured almost exclusively in participants with a high DNS score (1.4% vs. 0.3%). The effect size of the between-group difference was moderate to strong (Phi-coefficient = 0.225). A total of 22.0% of the participants with a low DNS score and 16.9% of the participants with a high DNS score stated that they never have their teeth cleaned professionally. Participants with a high DNS score were more likely to have their teeth cleaned twice a year compared to participants with a low DNS score (13.9% vs. 9.8%). However, there was no statistically significant difference between the participants with a low vs. high DNS score regarding the frequency of professional dental cleaning (*p* = 0.120).

## 4. Discussion

Based on the fact that, to our best knowledge, the DNS has not yet been used in German-speaking countries, the purpose of the present study was to evaluate the psychometric properties of a German version of the DNS. The psychometric properties of the DNS in our study can be classified as good, with a Conbach’s α = 0.710 and a McDonald’s ꞷ = 0.711. A number of studies have previously investigated the psychometric properties of this metric in relation to dental visits [8], caries susceptibility [14], and general health [8,14].

Our study, as well as a Romanian study [6], examined the convergent validity of the DNS through associations with other validated instruments. In our study, the DNS achieved significant negative correlations with the BSI-18 (Brief Symptom Inventory), OHIP-5 (Oral Health Profile), and DAS (Dental Anxiety Scale). The negative correlation confirms that people with higher psychological stress tend to show more dental neglect (i.e., have a lower DNS score). This agrees with previous findings such as the association of psychological distress with low self-care (including dental care) or avoidance behavior. Studies [40,41] that have already been conducted indicate that oral health significantly impacts overall, cognitive, and mental health, particularly among older adults. The ability to chew and maintain oral function is essential for ensuring a complete diet in the elderly. While most oral diseases are preventable, common issues like caries and periodontal disease remain prevalent. Neglecting the deterioration of oral function in older adults is common, which highlights the importance of early intervention and treatment. Collaborative efforts among politicians, dental associations, and researchers to deliver patient-centered, holistic care that integrates oral health into overall health management for improved wellbeing are recommended. The results of this study support the assumption that neglect of dental care is associated with poorer oral health-related quality of life (OHIP-5). The convergent validity of this study is moderate, but not strong. The correlation fits well to the theoretical expectations and shows that the DNS and OHIP-5 measures are related but not identical constructs. Other factors such as social determinants, genetic factors, and subjective perception of oral health seem to be important. This is further proof of the link between dental neglect and quality of life, psychological stress, and dental anxiety [5,6,15]. An exploratory factor analysis and a confirmatory factor analysis were used to test the factorial structure of the DNS. Our findings fit well with studies from Romania and Norway [6,7], supporting the hypothesis of a unifactorial structure. The total score obtained for the DNS-GER in this study was M = 25.63 (SD = 4.02), which indicates a high level of dental care. In comparison to participants of non-German studies, the German participants achieved high scores. The participants of a Romanian study showed similar results [6]. The values of other studies range from 10.18 to 19.71 [9,14,17]. The elevated DNS values obtained herein may be associated with the structure of the statutory health insurance system in Germany. Approximately 17.8 million individuals possess supplementary dental insurance in addition to their statutory coverage [42]. This additional insurance reflects a personal commitment to receiving optimal dental care. In this study, there were no differences between gender regarding whether participants obtained ahigh or low DNS score. These findings can be compared to the result of a study in Hong Kong [15] and a sample of young people in Washington State, USA [8]. In our study, men and women were similarly distributed in both groups. They showed comparable patterns in dealing with their dental health. This contradicts common assumptions about men tending to visit the dentist less often or having poorer oral hygiene, which was supported by the findings of a study in New Zealand [4]. Age emerged as a factor that has a significant influence on dental neglect. In this study, younger people were found to be more likely to neglect their dental health compared to older people. The reasons for this could be lack of time, lack of awareness, lower health priority, or dental anxiety. With regard to education, our analysis shows that there is no significant correlation between educational level (A-Levels vs. no A-Levels) and dental negligence. The Chi-square of 0.552 that was obtained in this study is very low, and the high *p*-value of 0.457 indicates that the differences between the groups could be purely random. Contrary to expectations and previously conducted studies which confirmed the influence of educational level on oral-health-related behavior [9,14,17], we did not identify educational level as a factor that determined dental care behavior. Furthermore, our study investigated the relationship between oral health and the DNS. The results showed that dental neglect did not have a strong influence on periodontal health (PSI) in this sample, while individuals with better dental care had higher DMF_T and DMF_S scores. These findings indicate that regular dental visits lead to higher documentation and treatment of dental damage. To better understand the actual impact of dental neglect, long-term studies and a more detailed analysis of untreated caries are needed.

Limitations: This study took place in one dental clinic only. The number of respondents in the sample was 311. Larger numbers of people are required for future studies in order to substantiate the results of this study. In addition, the answers to the questionnaires reflect subjective perceptions of oral health. Indices such as the DMF_T/DMF_S or the PSI reflect long-term damage, while the DNS tends to measure current behavior. Despite these limitations, the DNS-GER is expected to be a promising tool for assessing behaviors related to oral care and regular dental visits. In the context of the pediatric population, it is essential that the Dental Neglect Scale (DNS) be specifically adapted to the needs and characteristics of German children and adolescents to ensure age-appropriate and culturally relevant evaluation and care planning. Although socio-economic variables such as income level, employment status, and insurance coverage were not included in the original data collection and, therefore, could not be analyzed in the present study, they represent potentially influential factors in understanding dental neglect. These variables should be incorporated into future research designs and assessment instruments to enable a more comprehensive analysis of behavioral determinants in oral health and dental neglect.

## 5. Conclusions

This study aimed to adapt the Dental Neglect Scale (DNS) to the German population. Other population groups of different nationalities were examined in previous studies. In our study, the DNS was used in a German-speaking country for the first time. Our findings suggest that the DNS-GER can be regarded as a validated instrument for recording attitudes and behaviors in relation to oral hygiene and oral health. The implementation and evaluation of the survey is simple and easy to understand thanks to it being short and clear questionnaire. The DNS has emerged as a reliable tool for future research and practical applications in economically and precisely evaluating dental care and preventive behavior. Future research should aim to expand the use of the DNS to pediatric populations, in order to adequately capture and address age-specific characteristics and the unique psychosocial needs of children and adolescents. Using the DNS, dental problems can be identified and understood in a more targeted manner, allowing preventive measures to be optimized and future challenges in dentistry to be effectively minimized.

## Figures and Tables

**Table 1:** Descriptive statistics of all items of the DNS-GER (N = 311).

Dental Neglect Item (German Translation)	M	SD	Skew.	Kurt.	α If ItemDeleted	Corrected Item-Total
**1. I keep up my home dental care.** (Ich achte auf meine häusliche Zahnpflege.)	4.50	0.82	−1.740	2.724	0.613	r = 0.684
**2. I receive the dental care I should.** (Ich erhalte die zahnärztliche Versorgung, die ich benötige.)	4.62	0.87	−2.706	7.174	0.696	r = 0.350
**3. I need dental care, but I put it off. (reversed)** (Ich benötige eine zahnärztliche Behandlung, aber schiebe sie auf.) (umgepolt)	3.97	1.42	−1.048	−0.381	0.708	r = 0.383
**4. I brush as well as I should.** (Ich putze meine Zähne so gut, wie ich es sollte.)	4.25	0.97	−1.320	1.423	0.634	r = 0.571
**5. I control snacking between meals as well as I should.** (Ich kontrolliere das Naschen zwischen den Mahlzeiten so gut, wie ich kann.)	3.55	1.34	−0.491	−0.877	0.715	r = 0.349
**6. I consider my dental health to be important.** (Ich halte meine Zahngesundheit für wichtig.)	4.71	0.68	−2.68	7.721	0.659	r = 0.549

Note: M—mean, SD—standard deviation; Skwe—skewness; Kurt—kurtosis.

**Table 2:** Result of the exploratory factor analysis.

Item	Factor 1
Item 1	0.873
Item 2	0.502
Item 3	0.621
Item 4	0.745
Item 5	0.486
Item 6	0.772
Explained Variance %	46.44

**Table 3:** Goodness-of-fit indices of the confirmatory factor model.

Model	χ^2^	df	*p*	χ^2^/df	CFI	TLI	RMSEA [CI]	SRMR
Default	14.452	9	0.107	1.606	0.975	0.959	0.064 [0.00–0.122]	0.045

**Table 4:** Correlations between DNS, BSI-18, OHIP-5, DMF_T, DMF_S, PSI, and DAS.

Measures	1	2	3	4	5	6
1. DNS	-					
2. BSI-18	r = −0.283 *p* < 0.001 **	-				
3. OHIP-5	r = −0.299 *p* < 0.001 **	r = 0.234 *p* < 0.001 **	-			
4. DMF_T	r = 0.132 *p* = 0.024 *	r = −0.030 *p* = 0.606	r = 0.123 *p* = 0.034 *	-		
5. DMF_S	r = 0.137 *p* = 0.020 *	r = −0.054 *p* = 0.358	r = 0.110 *p* = 0.059	r = 0.968 *p* < 0.001 **	-	
6. PSI	r = 0.066 *p* = 0.290	r = −0.070 *p* = 0.262	r = −0.33 *p* = 0.592	r = 0.346 *p* < 0.001 **	r = 0.351 *p* < 0.001 **	-
7. DAS	r = −0.278 *p* < 0.001 **	r = 0.335 *p* < 0.001 **	r = 0.342 *p* < 0.001 **	r = 0.046 *p* = 0.435	r = 0.002 *p* = 0.971	r = −0.001 *p* = 0.994

* *p* < 0.05= statistically significant, ** *p* < 0.001 statistically highly significant.

**Table 5:** Differences in DNS between gender, age, education, and oral health-related variables (PSI, DMF_T, DMF_S, dental anxiety).

	Total Group	Low DNS Score (≤27)	High DNS Score (>27)	Test Statistics	*p*-Value	Effect Size
		N = 158 (52.8%)	N = 147 (48.2%)			
**Gender N = 304**						
Male	129 (42.43%)	72 (48.6%)	57 (51.4%)	Chi-Quadrat = 2.494	*p* = 0.287	
Female	175 (57.57%)	85 (55.8%)	90 (44.2%)			
**Age N = 305**						
Young (until 49)	137 (44.9%)	98 (71.5%)	39 (28.5%)	Chi-Quadrat = 38.77	***p*** < 0.001 **	Phi = 0.357
Old (50+)	168 (55.1%)	60 (35.7%)	108 (64.3%)			
**Education N = 305**						
A-Levels	106 (34.75%)	58 (54.7%)	48 (45.3%)	Chi-Quadrat = 0.552	*p* = 0.457	
No A-Levels	199 (65.25%)	100 (50.3%)	99 (49.7%)			
**Oral Health**						
PSI	N = 258	N = 137	N = 121	T = −2.325 df = 256	***p*** = 0.021 *	Cohen’s d = −0.290
	M = 11.79 (SD = 5.35)	11.07 (53.27)	12.61 (5.34)			
DMF_T	N = 293	152	141	T = −2.791 df = 290.851	***p* = 0.006** *	Cohen’s d = −0.332
	M = 16.39 (SD = 8.32)	15.1 (8.6)	17.77 (7.8)			
DMF_S	N = 289	149	140	T = −2.281 df = 287	***p*** = 0.005 *	Cohen’s d = −0.332
	M = 57.36 (SD = 34.67)	51.58 (35.33)	63.23 (33.08)			
DAS	N = 303	158	145	T = 4.812 df = 249.770	***p*** < 0.001 **	Cohen’s d = 0.548
	M = 10.34 (SD = 3.97)	11.29 (4.23)	9.19 (3.35)			

* *p* < 0.05 = statistically significant, ** *p* < 0.001 statistically highly significant.

**Table 6:** DNS and preventive behavior.

	N (%)		Chi-Square	*p*-Value	Effect Size
	Low DNS Score (<=27)	High DNS Score (>27)			
**How often do you brush your teeth each day?**					
1x	36 (11.9%)	12 (4.0%)	Χ^2^ = 14.112 df = 3	***p*** ** = 0.003**	Phi = 0.216
2x	106 (35.0%)	119 (39.3%)			
3x	11 (3.6%)	15 (5.0%)			
4x	3 (1.0%)	1 (0.3%)			
**How often do you go to the dentist per year?**					
**Never**	12 (4.0%)	1 (0.3%)	Χ^2^ = 15.046 df = 5	***p*** ** = 0.010**	Phi = 0.224
1x	72 (24.0%)	59 (19.7%)			
2x	47 (15.7%)	62 (20.7%)			
3x	6 (2.0%)	10 (3.3%)			
4x	3 (1.0%)	5 (1.7%)			
**More Often**	14 (4.7%)	9 (3.0%)			
**How many times do you have tartar removed per year?**					
**Never**	36 (12.2%)	12 (4.1%)	Χ^2^ = 16.480 df = 5	***p*** ** = 0.006**	Phi = 0.225
1x	74 (25.1%)	74 (25.1%)			
2x	38 (12.9%)	50 (16.9%)			
3x	1 (0.3%)	4 (1.4%)			
4x	2 (0.7%)	3 (1.0%)			
**More Often**	0 (0.0%)	1 (0.3%)			
**How often do you have your teeth professionally cleaned per year?**					
**Never**	65 (22.0%)	50 (16.9%)	Χ^2^ = 7.327 df = 4	*p* = 0.120	
1x	54 (18.2%)	42 (14.2%)			
2x	29 (9.8%)	41 (13.9%)			
3x	2 (0.7%)	6 (2.0%)			
4x	3 (1.0%)	4 (1.4%)			

## Data Availability

The data presented in this study are available on request from the corresponding author. The data are not publicly available due to privacy issues.
